# Physical activity of mice on dietary sulfur amino acid restriction is influenced by age of diet initiation and biological sex

**DOI:** 10.1038/s41598-023-47676-7

**Published:** 2023-11-23

**Authors:** Diana Cooke, Gene P. Ables

**Affiliations:** Orentreich Foundation for the Advancement of Science, Inc., 855 Route 301, Cold Spring, NY 10516 USA

**Keywords:** Ageing, Preclinical research, Skeletal muscle, Nutrition, Molecular biology

## Abstract

Sulfur amino acid restriction (SAAR)—the reduction of methionine and cysteine concentrations either in the diet or by genetic manipulation—promotes health span and extends lifespan, but its effects on physical activity remain unclear. We investigated whether age of diet initiation and biological sex could influence physical activity in mice fed either a control diet (CF, 0.86% methionine w/w) or SAAR (0.12% methionine w/w). Quadriceps femoris muscle mass is smaller in SAAR than in CF mice. Young mice fed a chronic SAAR diet at 8 weeks of age exhibited improved wire hang and running wheel activities compared to young CF mice, while aged mice showed comparable results. The effects of chronic SAAR on physical activity was mildly influenced by sex as observed in middle-aged male SAAR mice who showed minor improvements than CF males while middle-aged females displayed no discernible effects. Muscle mass is minimally affected by changes in markers of protein synthesis, autophagy and atrophy. Improvements to physical activity in young SAAR mice could be partially attributed to increased skeletal muscle mitochondrial activity. Furthermore, SAAR in C2C12 myotubes increased citrate synthase protein expression and enhanced succinyl dehydrogenase enzyme activity compared to CF myotubes. Overall, our data reveal that SAAR can improve mouse physical activity without compromising muscle proteostasis. This is partially due to enhanced mitochondrial activity, but the effects are influenced by age of diet initiation and sex.

## Introduction

As the aged human population (> 65 years old) continues to grow, it is vital to develop interventions that will extend healthy aging^1,2^. One potential intervention is sulfur amino acid restriction (SAAR), which reduces methionine and cysteine concentrations either in the diet or by genetic manipulation. SAAR has been shown to extend lifespan in rodents, fruit flies, and yeast^3–6^. It also promotes health benefits in mammals, such as preventing diabetes, obesity, and cancer^7–9^. In addition to preventing weight gain and fat accumulation, SAAR also results in shorter body length, smaller bones and lower lean mass^10–12^. However, it has not been tested whether the small size induced by SAAR affects physical activity.

The decline of physical activity in mice with age and its influence by biological sex is well-established^13,14^. For instance, aged male and female mice have reduced exercise capacity and distance run compared to their younger counterparts^13,15^. Additionally, grip strength is higher in aged females than aged males, while aged males run slightly greater distances than aged females^13,15^. However, the effects of age and sex on physical activity during SAAR conditions have not been investigated.

A number of studies in different species have investigated the molecular effects of sulfur amino acid restriction (SAAR) on skeletal muscle. In dogs, SAAR depleted taurine concentrations in skeletal muscle but not in plasma and blood^16^. In skeletal muscles of growing pigs, SAAR showed minimal changes in gene expression of myogenic factor 6 and myocyte enhancer factor 2D^17^. In a mouse model of skeletal muscle denervation and overload, SAAR aggravated atrophy but enhanced hypertrophy^18^. In rats, SAAR increased skeletal muscle mitochondrial oxidative activity and preserved mitochondrial function^19,20^. However, none of these studies assessed physical performance.

We investigated the impact of the initiation of SAAR on physical activity and how it is affected by age and sex in young, middle-aged, and aged male and female mice fed either a control (CF) or SAAR diet. We used wire hang for strength and endurance, rotarod for motor coordination and balance, and voluntary running wheel for physical activity. Our data revealed that initiating SAAR was most beneficial in young mice compared to aged mice, while both middle-aged male and female mice exhibited similar effects. We also propose that enhanced skeletal muscle mitochondrial activity may be a potential mechanism through which SAAR improves physical activity in young mice.

## Results

### Initiating SAAR in young male mice improves physical activity but not in aged male mice

**Figure 1:**
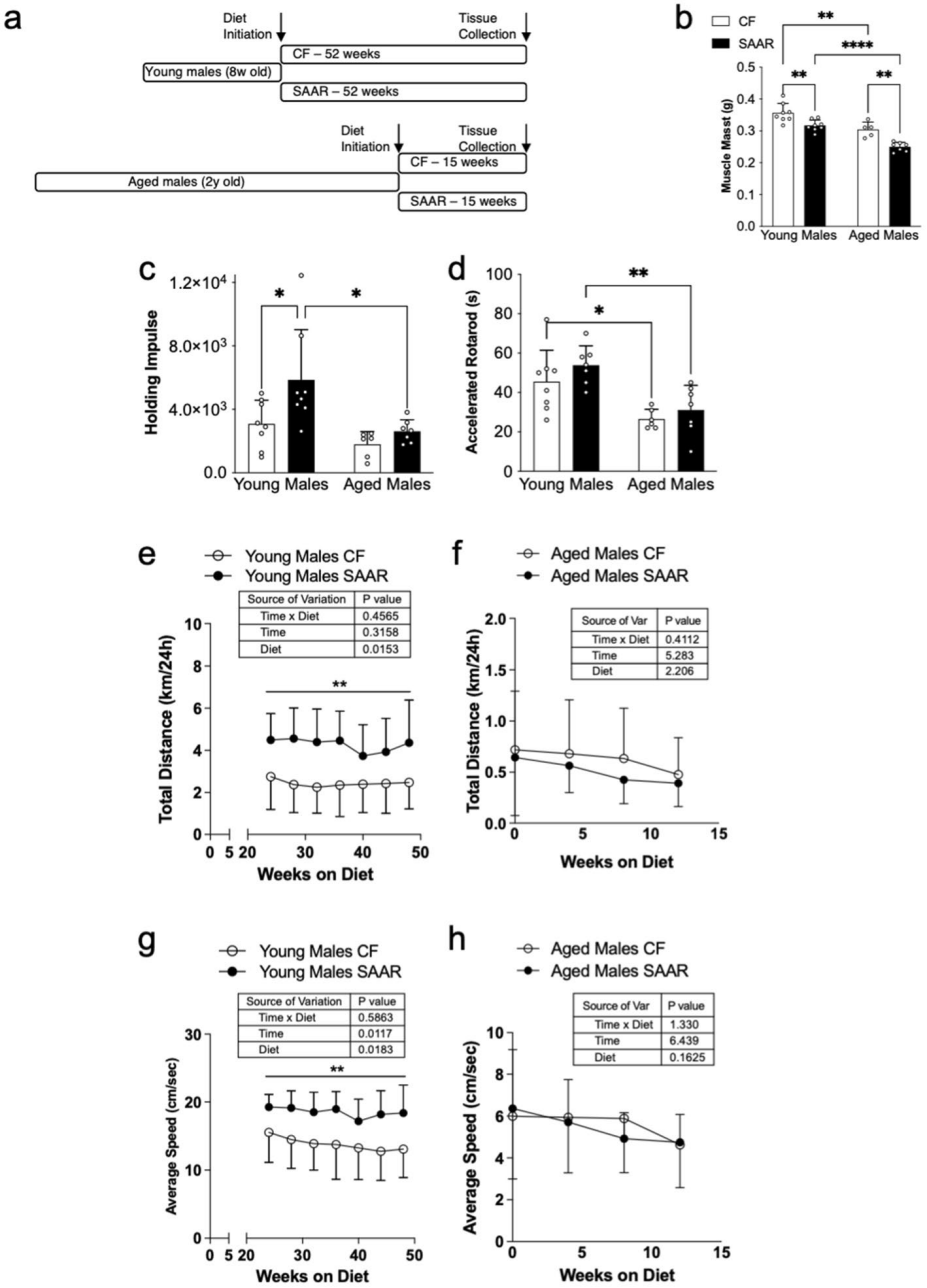
Physical activity of SAAR mice is influenced by age. (**a**) Young male mice were defined as 8 weeks old and fed either control (CF, 0.86% met w/w, white bars or circles) or SAAR (0.12 met w/w, black bars or circles) for 52 weeks. (**a**) Aged male mice were defined as 2 years old and fed either diet for 15 weeks. White dots within bar graphs indicate values from each animal. Muscle mass (**b**) was obtained from the quadriceps femoris of each mouse. Physical activity was assessed by wire hang shown as holding impulse (**c**), and accelerated rotarod (**d**) were performed 2 weeks prior to sacrifice. Distance run (**e** and **f**) and speed (**g** and **h**) from 24-h in-cage voluntary running wheel measurements collected every 2 weeks after 6 months on the diets. Statistical analysis was conducted using 2-way ANOVA with Sidak post hoc test, as described in methods (n = 5–8 per group, ^*^P < 0.05, ^**^P < 0.01, ^****^P < 0.0001).

To understand how SAAR initiation impacts physical activity, we studied its effects on skeletal muscle in both young and aged male mice. We classify young mice as those introduced to the diet at 8 weeks of age, exposed to either a control (CF) or SAAR diet for 52 weeks, thereby mitigating short-term diet-related effects (Fig. 1a). Aged mice are defined as those that began the diet at 2 years of age and were fed either diet for 15 weeks (Fig. 1a). Our data indicate that muscle mass was lower in young and aged male SAAR mice compared to CF counterparts (Fig. 1b, young SAAR = 0.317 ± 0.02 g; young CF = 0.356 ± 0.03 g, *P* < 0.01; aged SAAR = 0.25 ± 0.01 g; aged CF = 0.304 ± 0.02 g, *P* < 0.01). Aged male mice on both diets had lower muscle mass than young male mice (Fig. 1b, 2-way ANOVA, CF = *P* < 0.01, SAAR = *P* < 0.001). Consistent with the metabolic effects of SAAR^11^, young male SAAR mice exhibited lower weight gain, while aged male SAAR mice experienced weight loss compared to CF counterparts, despite consuming more food per gram of body weight (Supplementary Figures S1a–S1d).

**Figure 2:**
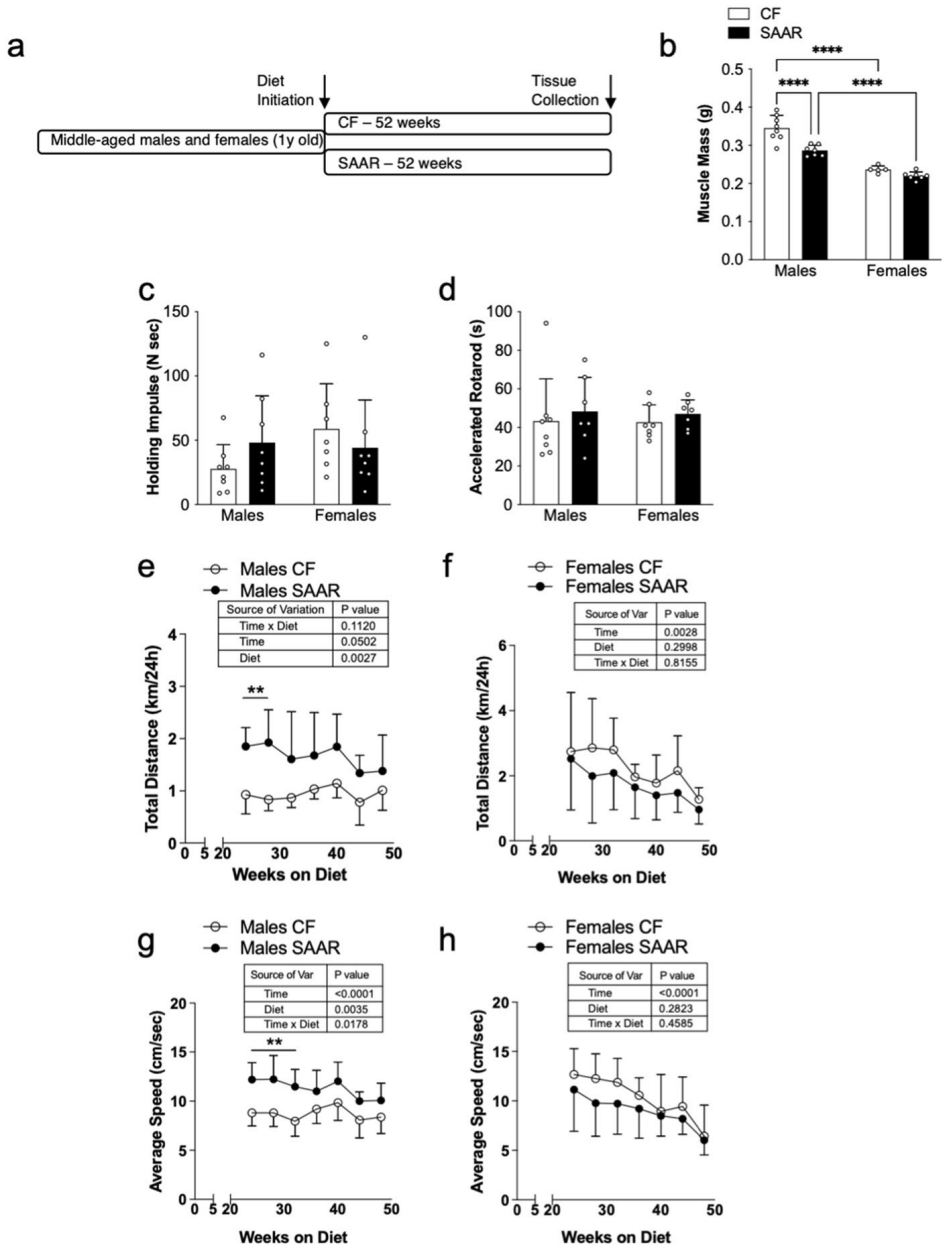
Physical activity of SAAR mice is minimally influenced by sex. (**a**) Middle-aged mice were defined as 1-year-old male and female mice and were fed either a control (CF, 0.86% met w/w, white bars or circles) or SAAR (0.12 met w/w, black bars or circles) diet for 52 weeks. White dots within bar graphs indicate values from each animal. Muscle mass (**b**) was obtained from the quadriceps femoris. Physical activity was assessed by wire hang and shown as holding impulse (**c**) and accelerated rotarod (**d**) performed 2 weeks prior to sacrifice. Distance run (**e** and **f**) and speed (**f** and **g**) from 24-h in-cage voluntary running wheel measurements collected every 2 weeks after 6 months on the diet. Statistical analysis was conducted using 2-way ANOVA with Sidak post hoc test, as described in methods (n = 7–8 per group, ^*^P < 0.05, ^**^P < 0.01, ^****^P < 0.0001).

We next asked whether SAAR affected physical activity. Holding impulse, a measure to correct for body weight, was 1.9-fold higher in young male SAAR mice than young male CF while aged male mice on both diets were not different (Fig. 1c, young SAAR = 57.5 ± 30.9 N sec vs young CF = 30.3 ± 14.7 N sec, *P* < 0.05, aged SAAR = 31 ± 11 N sec vs aged CF = 21 ± 6 N sec, *P* = 0.05). These wire hang data observed in young and aged mice were similar even when not corrected for body weight (Supplementary Figure S2a, young SAAR = 227 ± 127 secs vs young CF = 90 ± 53, *P* < 0.001, aged SAAR = 104 ± 40 secs vs aged CF = 55 ± 17 secs, ns). Due to a notable increase in wire hang ability among young male SAAR mice, a substantial decline was observed in aged male SAAR mice (Fig. 1c and Supplementary Figure S2a). Interestingly, the difference in holding impulse between young and aged CF male mice was not significant. (Fig. 1c and Supplementary Figure S2a). There was no significant difference in the latency to fall from rotarod in CF and SAAR male mice of either age group (Fig. 1d; young SAAR = 54 ± 10 secs vs young CF = 46 ± 16 secs, aged SAAR = 31 ± 12 secs vs aged CF = 27 ± 5 secs) but aged male mice on both diets fell faster than young male mice (Fig. 1d, 2-way ANOVA, CF = *P* < 0.05, SAAR = *P* < 0.01).

Voluntary exercise data indicate that young male SAAR mice ran significantly greater distances than young male CF mice at each test period (Fig. 1e), while aged male mice on both diets ran similar distances (Fig. 1f); diet as the main source of variation in young male mice (*P* < 0.05, F_1, 14_ = 7.629). The average distance for all time points is greater in young male SAAR mice than young male CF, while the averages in aged males were comparable (Supplementary Figure S2b, SAAR = 4.3 ± 1.3 km/24 h vs CF = 2.4 ± 1.4 km/24 h, *P* < 0.01, aged SAAR = 0.5 ± 0.3 km/24 h vs aged CF = 0.6 ± 0.5 km/24 h, *P* = 0.9). In addition, young male SAAR mice ran faster than young male CF mice at all time points (Fig. 1g) while aged male mice on both diets ran at similar speeds (Fig. 1h). The average speed for all time points was higher in young male SAAR mice than young male CF while the averages in aged males were comparable (Supplementary Figure S2c, young SAAR = 19 ± 2.6 cm/s vs young CF = 14 ± 4.2 cm/s, *P* < 0.05, aged SAAR = 5.4 ± 1.7 cm/s vs CF = 5.6 ± 2.5 cm/s). Diet and time were the sources of variation in young male mice (Fig. 1g, Diet: *P* < 0.05, F_1,14_ = 7.1, Time: *P* < 0.05, F_3,41_ = 4.14). Taken together, our results demonstrate that initiating chronic SAAR in young mice improve physical activity compared to initiating SAAR in aged male mice.

### Chronic SAAR in middle-aged mice minimally affects physical activity

Next, we asked whether the effects of SAAR on physical activity are influenced by biological sex. We initiated CF or SAAR diets in middle-aged male and female mice, who were 1 year old, and fed them these diets for an additional year to minimize the effect of short-term dietary effects (Fig. 2a). Our data show that SAAR reduced muscle mass in middle-aged males, but not in middle-aged females compared their CF counterparts (Fig. 2b, males SAAR = 0.286 ± 0.01 g; CF = 0.345 ± 0.03 g and, females SAAR = 0.22 ± 0.01 g; CF = 0.237 ± 0.01 g). In addition, middle-aged females on both diets had lower muscle mass than middle-aged males (Fig. 2b). Middle-aged male CF gained weight 14 days after diet initiation which was maintained at around 40 g until termination while male SAAR lost weight which was maintained at 27 g until termination (Supplementary Figure S1e). There was no significant difference between body weights of middle-aged female CF and SAAR mice (Supplementary Figure S1g). Food intake per gram of body weight was higher in both middle-aged male and female SAAR mice compared to their CF counterparts (Supplementary Figures S1f. and S1h).

In terms of physical activity, holding impulse, rotarod, and wire hang metrics showed similarity between middle-aged males and females on both diets (Fig. 2c,d, and Supplementary Figure S2d, male SAAR = 200 ± 146 secs vs male CF = 74 ± 49 secs; female SAAR = 188 ± 133 secs vs female CF = 221 ± 143 secs). Data from voluntary exercise showed that middle-aged male SAAR mice ran farther than male CF mice, with statistical significance observed only at the early time points (Fig. 2e), while middle-aged female mice ran comparable distances irrespective of their diet (Fig. 2f). There was no significant difference in the average distance run in middle-aged males and females on either diet (Supplementary Figure S2e, male SAAR = 1.7 ± 0.5 km/24 h vs male CF = 0.9 ± 0.2 km/24 h; female SAAR = 1.7 ± 0.9 km/24 h vs female CF = 2.2 ± 0.8 km/24 h). Interestingly, middle-aged female CF mice had greater average distance run than middle-aged male CF (Supplementary Figure S2e, *P* < 0.01). The main sources of variation based on total distance run at each time point was diet for middle-aged males (Fig. 2e, Diet: *P* = 0.0027, F_1,14_ = 13.2) and time for middle-aged females (Fig. 2f, Time: *P* = 0.0028, F_1,24_ = 7.9). In terms of speed, middle-aged male SAAR mice outpaced their controls, with significance observed mainly at early time points (Fig. 2g), while middle-aged females showed consistent speeds on both diets (Fig. 2h). The main source of variation for middle-aged males was interaction of time and diet (Fig. 2g, males, Time × Diet *P* = 0.0178, F_6,82_ = 2.7), whereas for middle-aged females, it was only time (Fig. 2h, Time: *P* < 0.001, F_2,33_ = 18.2). Average speed from all testing periods were similar for middle-aged males and females on both diets (Supplementary Figure S2f., male SAAR = 11.3 ± 1.5 cm/s vs male CF 8.7 ± 1.3 cm/s; female SAAR = 8.9 ± 2.4 cm/s vs female CF = 10.7 ± 1.9 cm/s). Our data demonstrate a progressive decline in physical activity with age, with a more pronounced decrease in females compared to males. Taken together, our data reveal that initiating chronic SAAR in middle-aged mice has minimal impact on physical activity in males, with no adverse effects observed in females.

### Dietary SAAR altered circulating profile in mice

Next, we measured circulating biomarkers that are common during SAAR (Table 1)^7^. Our data indicate that circulating levels of glucose, insulin, leptin, and IGF-1 were reduced in young and aged SAAR mice compared to CF counterparts. In addition, adiponectin and FGF21 concentrations were elevated in young and aged SAAR mice compared to their CF counterparts. Interestingly, we observed a significant decline in concentrations of adiponectin and FGF21 in aged SAAR mice compared to young SAAR mice.

**Table 1:** Circulating biomarkers of mice on CF and SAAR diets.

	Young		Aged		Males		Females	
	CF	SAAR	CF	SAAR	CF	SAAR	CF	SAAR
Glucose (mg/dl)	116 ± 13	94 ± 14**	112 ± 10	92 ± 11**	117 ± 22	102 ± 13	62 ± 21^*b*^	69 ± 21^*c*^
Insulin (ng/ml)	1.3 ± 0.6	0.4 ± 0.2**	1.4 ± 0.3	0.6 ± 0.3**	1.6 ± 0.4	0.7 ± 0.2***	1.2 ± 0.8	0.7 ± 0.3
Leptin (ng/ml)	24.8 ± 17.5	4.2 ± 2.4**	24.0 ± 7.4	6.1 ± 1.8*	33.7 ± 15.6	3.7 ± 1.5***	13.3 ± 7.3^*b*^	4.2 ± 1.8**
IGF-1 (ng/ml)	331 ± 49	213 ± 34***	280 ± 48	194 ± 23**	454 ± 98	237 ± 25***	285 ± 35^*a*^	260 ± 59
Adiponectin (µg/ml)	8.8 ± 0.7	12.1 ± 0.7***	8.3 ± 0.8	10.3 ± 1.1**^,*a*^	9.2 ± 0.8	10.1 ± 0.7	14.0 ± 4.3^*b*^	14.1 ± 1.9^*c*^
FGF21 (ng/ml)	1.1 ± 0.4	4.4 ± 1.2***	0.7 ± 0.2	2.3 ± 1.0*^,*a*^	0.8 ± 0.5	2.6 ± 1.0**	0.4 ± 0.2	1.8 ± 1**

Comparison between diets in the same age and sex were analyzed using two-tailed unpaired Student’s t-test (n = 5–8 / group, *P < 0.05, **P < 0.01, ***P < 0.001). Comparisons between young and aged male, as well as between middle-aged male and female mice on either diet were analyzed by 2-way ANOVA described in Methods.

^a^SAAR values are significantly different from young SAAR.

^b^CF females were significantly different from CF males.

^c^SAAR females were significantly different from SAAR males, *P* < 0.05). IGF-1, insulin-like growth factor-1; FGF21, fibroblast growth factor-21.

Biological sex influenced the effect of SAAR on circulating biomarkers. Blood glucose was significantly lower in middle-aged females than in middle-aged males, regardless of diet (Table 1). Insulin and IGF-1 were reduced in middle-aged male SAAR compared to CF counterparts but were unchanged in middle-aged females, with female CF having lower values male CF. Leptin was reduced in middle-aged male and female SAAR mice compared to CF counterparts, with female CF having lower values than male CF. Adiponectin were comparable in middle-aged male and female mice on both diets, but females had higher concentrations than males. FGF21 was elevated in middle-aged male and female SAAR mice compared to CF counterparts. Collectively, our data reveal that biomarker concentrations during SAAR in mice are more influenced by biological sex rather than by age.

### SAAR induced minor changes in proteostasis markers in muscle

**Figure 3:**
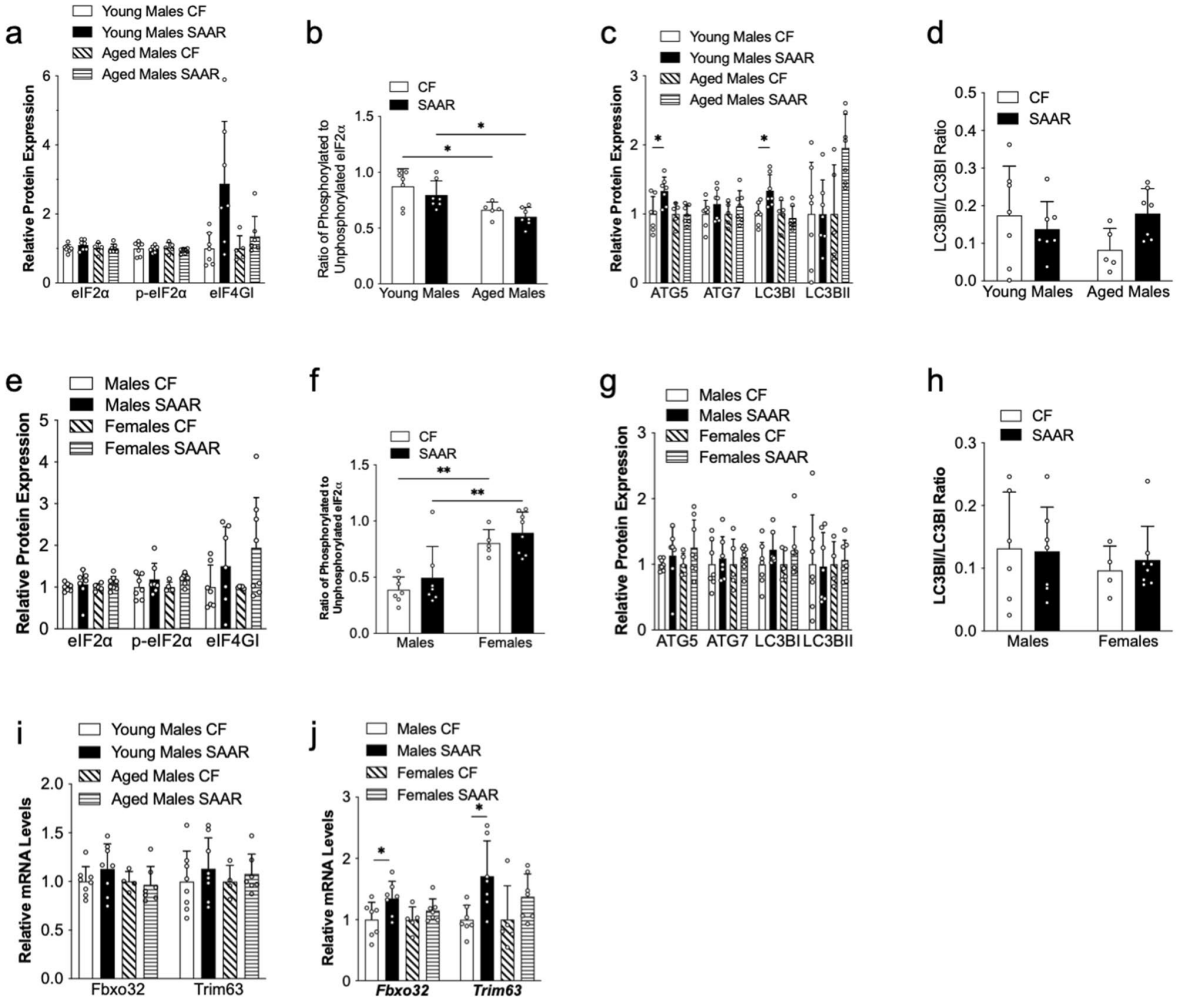
The effects of SAAR on mouse muscle proteostasis is minimally affected by age of diet initiation and biological sex. Muscle tissues (quadriceps femoris) were collected at the end of diet intervention from young mice, which were fed the diet at 8 weeks old for 1 year; aged mice, which were fed the diet at 2 years old for 15 weeks; and middle-aged male and female mice, which were fed the diet at 1 year old for 52 weeks. These mice were given either a control diet (CF, 0.86% methionine w/w) or an SAAR diet (0.12% methionine w/w), as outlined in the Methods section. Proteins and RNA were extracted, and markers related to protein synthesis, autophagy, and atrophy were measured using Western blot and quantitative PCR, respectively, as outlined in the Methods section. White dots within bar graphs indicate values from each animal. Band intensities for each protein was normalized to Ponceau S stain from the same membrane from young and aged males (**a** and **c**), and middle-aged males and females (**e** and **g**). The ratios of phosphorylated to total proteins of eIF2α (**b** and **f**), and LC3BII to LC3BI (**d** and **h**) in muscles from CF and SAAR mice were quantified as described in Methods. Original membrane blots are presented in Supplementary Figure S3 and S4. Expression of atrophy related genes *Fbxo32* and *Trim63* from muscles of young and age (**i**) and middle-aged male and female mice (**j**) fed either a CF or SAAR diets. Comparisons between diets in the same age and sex were analyzed using Student’s t-test, as described in Methods (n = 5–8 per group, ^*^P < 0.05, ^****^P < 0.0001).

To gain mechanistic insight on reduced muscle mass in SAAR mice, we asked whether protein homeostasis is affected by examining protein synthesis, atrophy, and autophagy signaling pathways. We emphasize that tissues were collected after one year feeding in young and middle-aged mice, ensuring that the data reflect the effects of chronic intervention. In protein synthesis, we observed comparable eIF2α protein expressions in both total and phosphorylated forms in muscles of CF and SAAR mice in all cohorts (Fig. 3a,e). The eIF4G1 expression increase in young male SAAR mice compared to young male CF mice lacks statistical significance, and there is no significant expression change in aged males, middle-aged males, or female mice on both diets (Fig. 3a,e). The phosphorylated-to-total eIF2α ratio remained consistent for CF and SAAR muscles in young and aged male mice, but decreased in aged males (Fig. 3b); similarly, this ratio was stable in middle-aged males and females, yet increased in female CF and SAAR mice compared to males (Fig. 3f). Immunoblots for each protein are indicated in Supplementary Figure S3 and S4.

We proceeded to investigate the role of autophagy in maintaining muscle mass during SAAR. Autophagy is essential for muscle mass preservation^21,22^ and its activation is a potential mechanism through which SAAR may enhance health span^6,7,23^. Our results demonstrate elevated expressions of autophagy-related protein 5 (ATG5) and microtubule-associated protein 1 light chain 3 B I (LC3BI) in the skeletal muscles of young male SAAR mice when compared to young male CF mice, with consistent levels observed in aged males; meanwhile, the expression of LC3BII remained unaltered across both diet groups in all cohorts (Fig. 3c). We did not detect any changes in autophagy markers in middle-aged male and female on both diets (Fig. 3g). The ratios of LCBII to LC3BI, a marker of autophagic flux, were comparable in both groups of young and aged mice as well as in middle-aged male and female mice (Fig. 3d,h, respectively). Immunoblots for each protein are indicated in Supplementary Figure S3 and S4. Collectively, our data suggest that skeletal muscle proteostasis is minimally affected by chronic SAAR regardless of age of diet initiation or biological sex.

We then assessed the expression of atrophy-related genes to ascertain their contribution to reduced muscle mass in SAAR mice. Our data demonstrate similar gene expressions of F-box protein 32 (*Fbxo32*) and tripartite motif-containing 63 (*Trim63*) in both young and aged male CF and SAAR mice (Fig. 3i). Nonetheless, *Fbxo32* and *Trim63* were upregulated in middle-aged male SAAR mice compared to middle-aged male CF mice, while there were no changes in females (Fig. 3j).

### The effects of SAAR on muscle mitochondrial activity

**Figure 4:**
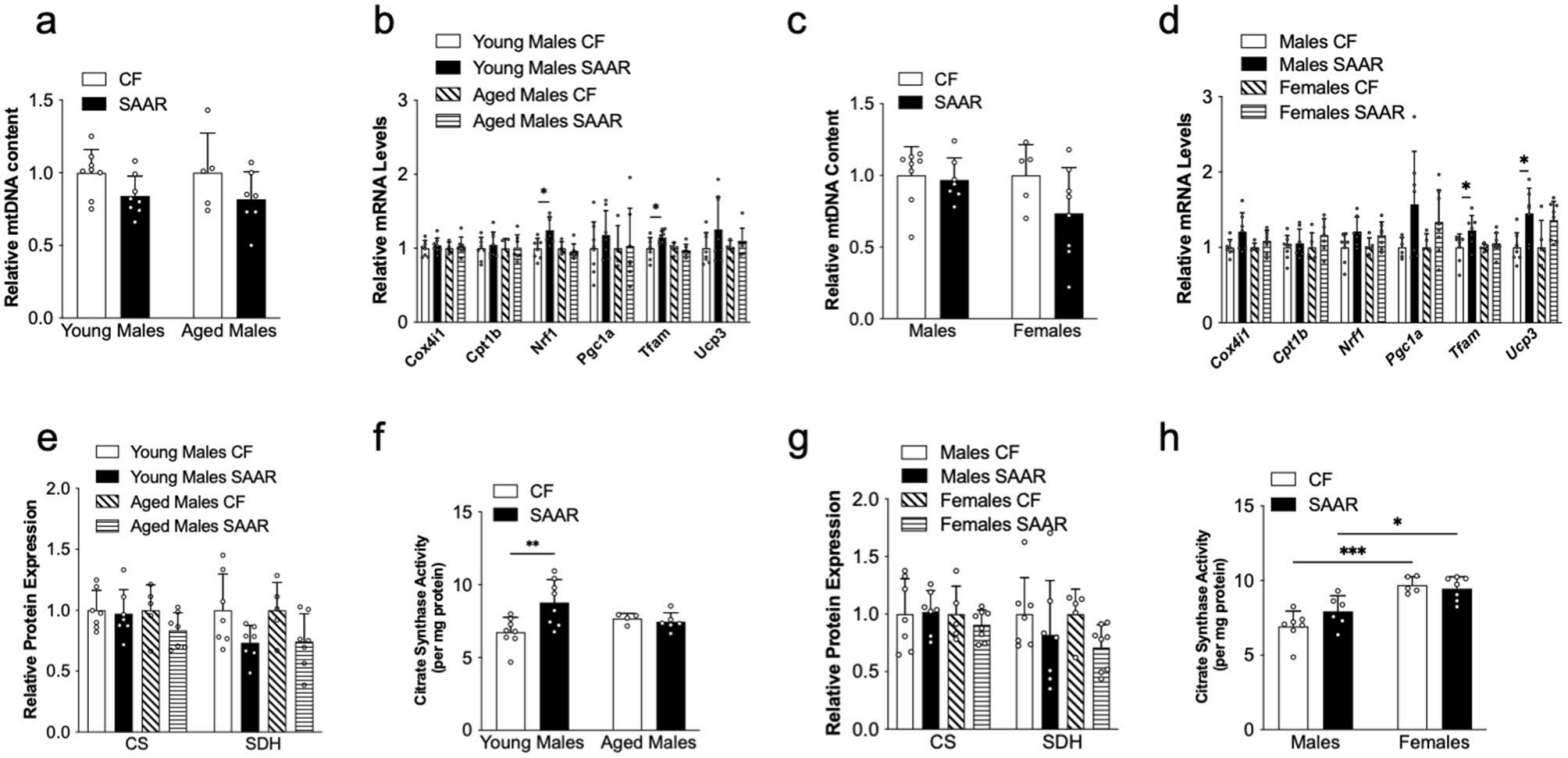
The effects of SAAR on mouse muscle mitochondrial activity is affected by age of diet initiation and biological sex. Muscle tissues (quadriceps femoris) were collected at the end of diet intervention from young male mice, which were fed the diet at 8 weeks old for 1 year; aged male mice, which were fed the diet at 2 years old for 15 weeks; and middle-aged male and female mice, which were fed the diet at 1 year old for 52 weeks. These mice were given either a control diet (CF, 0.86% methionine w/w) or an SAAR diet (0.12% methionine w/w), as outlined in the Methods section. White dots within bar graphs indicate values from each animal. Mitochondrial DNA (mtDNA) was isolated from muscle tissues of young and aged male (**a**), and middle-aged male and female (**c**) mice and its expression was normalized relative to CF counterparts. Genes linked to mitochondrial function were quantified via real-time PCR in muscle tissues of both young and aged (**b**) mice, as well as in middle-aged male and female (**d**) mice. Protein levels of citrate synthase and succinyl dehydrogenase (CS and SDH, respectively) were determined through Western blotting in muscle tissues of young and aged males (**e**) and middle-aged males and females (**g**) mice, with original blots in Supplementary Figures S3 and S4. Citrate synthase activity was assessed using an enzymatic assay, as detailed in the Methods section, in muscle tissues from both young and aged males (**f**), as well as from middle-aged males and female (**h**) mice. mRNA and protein levels were quantified relative to CF within each age or sex group and subsequently analyzed using a t-test, as detailed in the Methods section (n = 5–8 per group, ^*^P < 0.05, ^**^P < 0.01, ^***^P < 0.001).

To unravel the mechanism underlying SAAR's impact on physical activity, we investigated muscle mitochondrial activity. Prior research has demonstrated that SAAR enhances skeletal muscle mitochondrial oxidative activity^18,20^. Our data indicate no differences in relative mitochondrial DNA content in any of the tested cohorts on either diet (Fig. 4a,c). In young male SAAR mice compared to young male CF mice, gene expression analysis showed increased levels of nuclear respiratory factor 1 (*Nrf1*) and mitochondrial transcription factor A (*Tfam*), indicating enhanced mitochondrial activity, which remained unaltered in aged male mice (Fig. 4b). Middle-aged male SAAR had higher expression of *Tfam* and uncoupling protein 3 (*Ucp3*), an important regulator of thermogenesis in skeletal muscle, compared to male CF mice while both genes were unchanged in middle-aged female mice (Fig. 4d). Furthermore, other genes associated with mitochondrial function, such as cytochrome c oxidase subunit IV isoform 1 (*Cox4i1*), carnitine palmitoyltransferase 1b (*Cpt1b*), and peroxisome proliferator-activated receptor coactivator-1-alpha (*Pgc1a*), were comparable in diet all cohorts (Fig. 4b,d). Citrate synthase (CS) and succinyl dehydrogenase (SDHA) protein expressions, indicating intact mitochondria and mitochondrial oxidation, respectively, remained constant in both diets on all cohorts (Fig. 4e,g). Immunoblots for each protein are indicated in Supplementary Figure S3 and S4). Notably, young SAAR mice displayed higher citrate synthase enzyme activity compared to young CF mice (Fig. 4f). Interestingly, females on both diets showed greater citrate synthase activity than males (Fig. 4h). Collectively, our data suggest that SAAR in mice could increase mitochondrial activity, which is influenced by age but not by biological sex.

### Effects of SAAR on mouse muscle cell line

**Figure 5:**
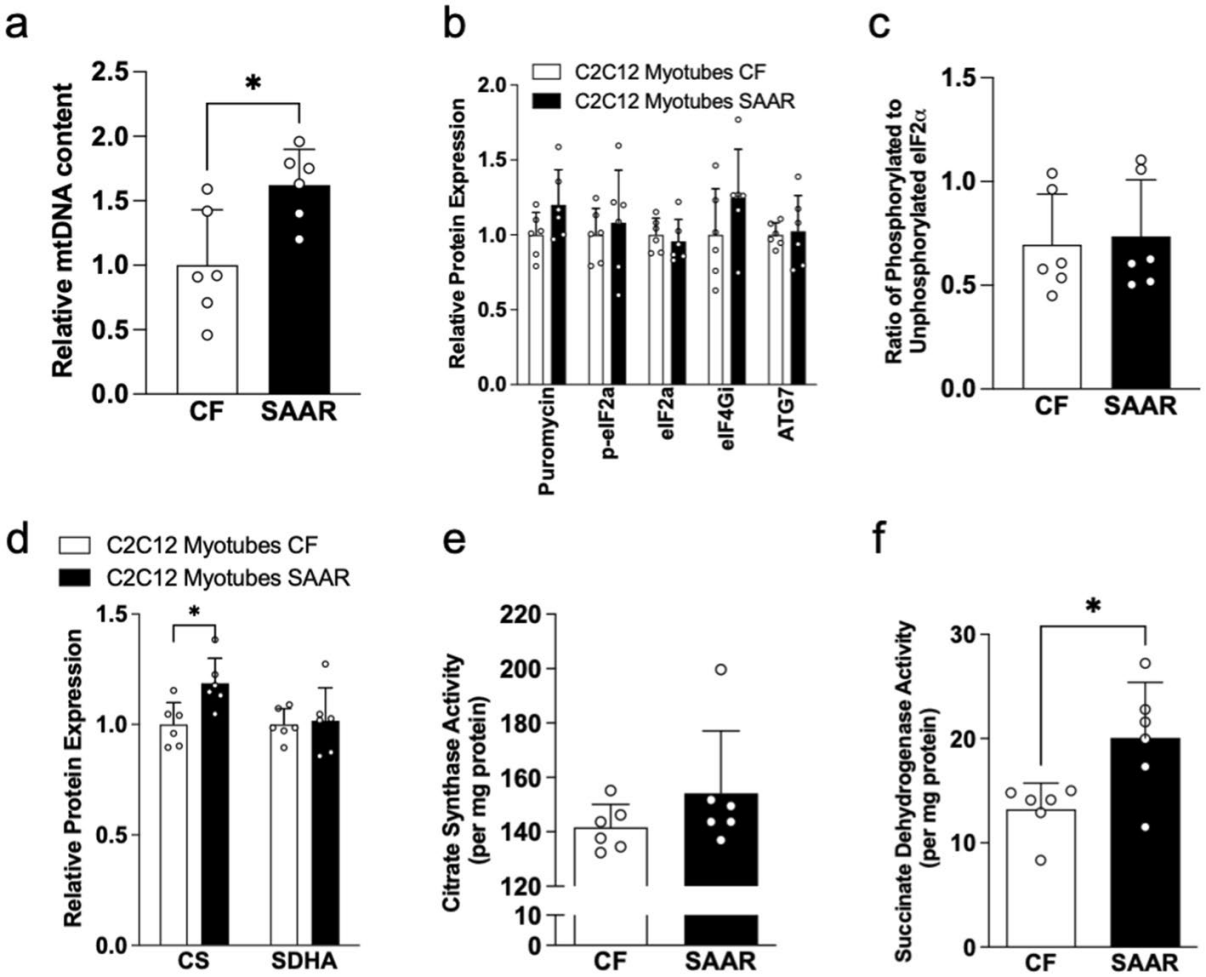
SAAR enhanced mitochondrial activity in C2C12 myotubes. Relative mitochondrial DNA content in differentiated C2C12 myotubes exposed to either CF (white bars) or 80% SAAR (black bars) media for 24 h (**a**) as described in Methods. White dots within bar graphs indicate values from each well. Proteostasis-related protein expressions, including puromycin, phosphorylated and total eIF2a, eIF4GI, and ATG7, were assessed in C2C12 myotubes exposed to either CF or SAAR conditions as described in Methods (**b**). The ratio of phosphorylated to unphosphorylated eIF2a in C2C12 myotubes was also determined (**c**). Furthermore, protein expressions of mitochondrial activity enzymes, such as citrate synthase (CS) and succinate dehydrogenase (SDHA), were measured in C2C12 myotubes exposed to either CF or SAAR conditions as detailed in Methods (**d**). Whole membrane immunoblots are presented in Supplementary Figure S5. CS and SDH activities (**e** and **f**, respectively) determined by enzymatic assay described in Methods. Comparisons between treatments were analyzed using Student’s t-test, as described in Methods (n = 6 per group; ^*^P < 0.05).

Next, we exposed C2C12 myotubes to 80% SAAR for 24 h to investigate its impact on muscle proteostasis and mitochondrial activity. Our results revealed significantly higher mtDNA content in SAAR myotubes compared to CF (*P* < 0.05). Global protein synthesis assessed by puromycin was similar in both cell types. Other proteostasis markers, such as eIF2a, eIF4GI, and ATG7 proteins, remained unchanged in both CF and SAAR myotubes (Fig. 5b), as did the ratio of phosphorylated to unphosphorylated eIF2a (Fig. 5c). Citrate synthase (CS) showed higher protein expression in SAAR myotubes compared to CF (*P* < 0.05), while succinyl dehydrogenase (SDHA) exhibited similar expressions in both groups (Fig. 5d). CS enzyme activity remained constant (Fig. 5e), while SDH activity increased in SAAR-treated myotubes (*P* < 0.05). In summary, our data indicate that SAAR directly influences mitochondrial activity with minimal impact on muscle proteostasis.

## Discussion

Our study investigates how age at initiation of diet and the biological sex of mice undergoing SAAR affect physical activity, and whether these effects are influenced by skeletal muscle proteostasis and mitochondrial activity. We demonstrate that SAAR in mice resulted in reduced muscle mass while improving physical activity, with age at initiation of diet and sex playing significant roles in these outcomes. Furthermore, skeletal muscles of SAAR mice exhibit subtle changes in proteostasis. The primary observation is that initiating chronic SAAR in young male mice results in superior strength and endurance compared to CF counterparts, which can be partially attributed to enhanced skeletal muscle mitochondrial activity. We corroborate our in vivo findings in C2C12 myotubes exposed to SAAR, which exhibited enhanced mitochondrial activity with minimal effects on proteostasis.

Mouse physical activity declines during aging^24–26^. Aged mice have lower grip strength, wire hang, rotarod, and voluntary running wheel measurements compared to young mice^24,25^. In addition, older male and female mice travel shorter distances, have slower gait speeds, and have reduced durations of locomotion, rearing, and climbing compared to young mice^26^. Because of this age-related deterioration, mouse physical activity has been assessed in other pro-longevity dietary interventions such as calorie, total protein, or branched-chain amino acid (BCAA) restriction^27–30^. Calorie-restricted mice have higher grip strength, and longer durations in wire hang and rotarod tests compared to mice fed ad libitum^29,30^. Mice undergoing protein restriction display no adverse effects of the diet on rotarod, wire hang and grip strength tests^27^. In addition, lifelong BCAA-restriction in male and female mice does not adversely affect rotarod activity or grip strength^28^. In terms of SAAR, physical activity of 12-month-old SAAR mice, expressed as beam breaks, is significantly higher than that of 12-month-old CF mice and is equivalent to that of 2-month-old CF mice^31^. We previously reported that HFD SAAR mice stay longer on a fixed rotarod compared to controls^12^. Our current data demonstrates that initiating chronic SAAR in young mice can enhance physical activity, while its initiation in aged and middle-aged mice does not yield adverse effects.

Similar to some of our current data, previous studies have established that the metabolic effects of SAAR in rodents are influenced by biological sex^11,32,33^. Forney et al. demonstrated that young male SAAR mice accumulate more lean mass, not fat mass, while young female SAAR mice accumulate fat mass, not lean mass, compared to CF counterparts^33^. They also showed that mature female SAAR mice have more robust energy expenditure than mature male SAAR mice compared to their CF counterparts^33^. In contrast, male SAAR mice have less weight gain and fat accumulation with concomitant increase in energy expenditure compared to CF males while those metrics were comparable in females^34^. We reported that SAAR initiation in 9-month-old male mice reduced body mass compared to CF counterparts, with no changes observed in age-matched female mice on either diet^11^ which is consistent with our current study. Collectively, these data reveal that the degree of SAAR in females is different than males to obtain optimum metabolic benefits. At the mechanistic level, it has been demonstrated that the effects by SAAR are partially influenced by sex. Studies showed that SAAR reduces the ratio of circulating S-adenosylmethionine to S-adenosylhomocysteine in female mice, but not in males, and increases the ratio of free reduced glutathione to oxidized glutathione in males but not in females^35^. We reported that circulating markers for bone metabolism such as C-terminal telopeptide of type 1 collagen is higher in middle-aged SAAR male than middle-aged CF while unchanged in females^11^. Additionally, receptor activator for nuclear factor κB ligand is decreased in adult SAAR females compared to adult CF females but unchanged in adult males^11^. In gut microbiome, Bacteroidaceae and Verrucoccaceae families were higher in SAAR male mice but lower in SAAR females compared to their CF counterparts^35^. In organ tissues, SAAR downregulated gene expressions of *Scd1* in the liver and *Lep* in white fat, and upregulated *Ucp1* in brown fat in male mice, but these changes were not observed in females^33^. In kidneys, SAAR upregulated *Fas*, *Srebp1c*, and *Acc1*, and downregulated *Pepck* in females but not in males^32^. Overall, we emphasize the importance of considering biological sex in dietary intervention studies.

Skeletal muscle mass can be maintained by several dynamic mechanisms, including protein synthesis, autophagy, and atrophy. A recent study showed that SAAR mice have smaller gastrocnemius, m. plantaris and m. soleus muscles than CF mice^18^. Congruent with these results, we also found that muscle mass was lower in SAAR mice compared to CF regardless of age at initiation. The evidence on whether SAAR affects protein synthesis in rodent skeletal muscle is remains unclear. For example, SAAR in mice suppressed protein synthesis in cytoplasmic fractions but not in mitochondrial fractions in gastrocnemius^36^. High fat diet (HFD) SAAR mice had slower skeletal muscle protein synthesis rates in mixed and cytosolic fractions compared to CF mice^37^. In contrast, protein synthesis rates were comparable in skeletal muscle of CF and SAAR rats^38^. At the mechanistic level, our data suggest that SAAR affected skeletal muscle protein synthesis differently from other studies. For instance, previous research has shown higher phosphorylation of eIF2α in the liver and skeletal muscles of SAAR mice compared to CF counterparts^34,36,37,39^, whereas in our cohorts, we observed no changes in this protein. In our in vitro experiments, we also did not observe major changes in the global protein synthesis rate, other proteostasis markers, or the ratio of p-eIF2a to eIF2a in SAAR C2C12 myotubes. Interestingly, the levels of hepatic eIF4G1 were lower in SAAR rats when compared to CF rats^38^, but we found a slight increase in skeletal muscle eIF4G1 protein levels in young male SAAR mice compared to young CF mice, but this difference was not statistically significant, and the same trend was observed in C2C12 myotubes exposed to SAAR. Collectively, our findings fail to provide substantial evidence supporting the notion that the decreased muscle mass in SAAR mice is linked to changes in protein synthesis.

We investigated the autophagy pathway in skeletal muscles because its activation is one of the mechanisms by which SAAR promotes the extension of lifespan^6,23^. Moreover, we documented a case of tissue-specific autophagy, where SAAR reduced fat mass among obese mice, partially due to activated macroautophagy in visceral fat and chaperone-mediated autophagy in inguinal fat^7^. In this connection, mice that lacked muscle-specific ATG7, an important component of autophagy, exhibited muscle degeneration due to the presence of abnormal mitochondria, accentuating that autophagy is required to maintain muscle mass^21^. Age and sex have had an impact on autophagy-related proteins in skeletal muscle, where Beclin1 exhibited higher levels in aged mice compared to young mice, and ATG7 showed higher levels in females than male mice^13^. Our data from skeletal muscles—indicating that initiating chronic SAAR in young mice increased protein expressions of ATG5 and LC3BI compared to CF mice, while no other protein was affected—suggest a minor role of autophagy in maintaining mass.

Muscle atrophy could also be affected by SAAR. The upregulation of atrophy-related genes *Fbxo32* and *Trim63* in middle-aged male SAAR mice could explain its smaller muscles. However, because these atrophy markers were unchanged in young and aged males and middle-aged females, it is highly possible that other mechanisms play a role in maintaining muscle mass during SAAR. Overall, our data indicate that the reduced muscle mass in SAAR mice does not negatively affect their physical performance.

Skeletal muscle mitochondrial activity is influenced by age and sex. For example, aged mice have lower skeletal muscle mitochondrial respiratory activity than young mice^13^. In addition, skeletal muscles of females have higher mitochondrial protein content from each complex of the electron transport chain than males^13^. Interestingly, skeletal muscle mitochondrial Complex-I protein is lower in aged males than in young males but higher in aged females than young females^13^. During SAAR, male rats display enhanced mitochondrial activity with increased citrate synthase activity and 3-hydroxybutyrate levels compared to CF^20,40^. In addition, SAAR had no adverse effects on mitochondrial function in rat skeletal muscle when measured for respiration, proton leak, and hydrogen peroxide metabolism, compared to CF^19^. Skeletal muscles of SAAR mice on HFD have higher SDH activity than control mice^18,41^. Our current study demonstrates that initiating chronic SAAR in young mice enhances skeletal muscle mitochondrial activity, which implies a potential connection with physical activity. Moreover, the heightened physical activity in young SAAR mice is corroborated by the absence of effects on physical and mitochondrial activities in aged mice, as well as middle-aged male and female mice. Our data indicate the need to optimize SAA requirements for the other cohorts to induce benefits. The ideal range for producing metabolic benefits in males was found to be between 0.17% to 0.25% methionine (w/w)^42^. Therefore, we recommend testing other dosages of SAAR while considering age of diet initiation and sex.

An unexpected result in our study was the increase in mtDNA in SAAR myotubes, which could potentially confound the interpretation of increased mitochondrial activity. However, our data in myotubes show that SAAR upregulated the expression of CS protein but not SDHA, while also increasing SDH activity but not CS. This suggests that mtDNA only plays a partial role in the enhanced mitochondrial activity during SAAR. Given that SAAR could enhance mitochondrial activity in myotubes, a tissue-specific induction may prove to be a promising approach.

Our study has some limitations. First, we utilized only quadriceps muscle, which has both fast- and slow-twitch types of muscle fibers. Examining the effects of SAAR on fast-twitch muscles like the gastrocnemius and slow-twitch muscles like the soleus separately, and testing muscle bioenergetics, would be relevant. Second, we were unable to test muscle contractile physiology, which declines in aged mice^24^, in our cohorts. We postulate that the effects of SAAR on muscle contractile physiology will be influenced by age and sex, and strongly recommend these experiments in future studies. Furthermore, we did not include a group of young female mice in this study because the effects of SAAR in them are similar to young males in terms of body weight and biomarkers^11^. Finally, we lack high-resolution respirometry data from mitochondria, which could have provided better insights into the effects of SAAR on mitochondrial activity. In summary, our research indicates that the initiation of chronic SAAR in young mice enhances physical activity, partly attributed to increased muscle mitochondrial activity, even in the presence of reduced muscle mass. We recommend a more comprehensive study on the muscle-specific effects of SAAR on mitochondria.

## Materials and methods

### Animals

All experiments were approved by the Institutional Animal Care and Use Committee of the Orentreich Foundation for the Advancement of Science, Inc. (Permit 0511 MB) and follow the recommendations in the ARRIVE guidelines. All methods were conducted in accordance with international ethical standards and guidelines. C57BL/6J (Catalog # 000664) mice were purchased from The Jackson Laboratory (Bar Harbor, ME, USA). Upon arrival, each mouse was singly housed with nestlets, maintained at 20 ± 2 °C and 50 ± 10% relative humidity on a 12 h light: 12 h dark photoperiod with ad libitum food (Laboratory Rodent Diet 5001, PMI Nutrition International, Brentwood, MO) and water. When the appropriate age was reached, mice were weight matched and fed either a control diet (CF; 0.86% methionine w/w) or SAAR (0.12% methionine w/w) diet that consisted of 14% kcal protein, 76% kcal carbohydrate, and 10% kcal fat (Research Diets, New Brunswick, NJ, USA; Supplementary Table S1). Body weight and food consumption were measured twice weekly. Young (8-week-old) male mice were fed either CF (n = 8) or SAAR (n = 8) diet for 52 weeks and sacrificed at 60 weeks old. Aged (2-year-old) male mice fed either a CF (n = 5) or SAAR (n = 7) diet for 15 weeks and sacrificed at 2 years, 15 weeks old. Middle-aged (1-year old) male and female mice were fed either a CF (males n = 8, females n = 5) or SAAR (males n = 7, females n = 8) diet for 52 weeks and sacrificed at 2 years old. Data for body weights of male mice used in this study were published in a recent manuscript (Supplementary Figures S1a, S1c and S1e)^43^. During the course of the study, 2 out of 7 aged CF, 1 out of 8 middle-aged male SAAR, and 3 out of 8 female CF mice died before the end of the experiments due to undetermined causes. Mice that died before termination of the study were excluded in data analysis. On the day of sacrifice, mice were fasted for 4 h, then euthanized by CO_2_ asphyxiation. Plasma and muscle (Quadriceps femoris) were collected, snap frozen in liquid nitrogen, and stored at ‒ 80 °C until processed. Muscle tissues were collected 2 weeks after the last physical test to directly observe the effect of the diet.

### Physical activity tests

#### Wire hang test

Wire hang tests were conducted 2 weeks prior to sacrifice, as described previously^44^. Briefly, each mouse was placed on a wire lid and turned upside down at ~ 60 cm above a surface. Mice were conditioned on the wire lid for 2 days; data was collected on the third day. The latency to fall from the inverted wire was analyzed. Holding impulse, which represents the minimal force exerted to oppose gravitational force, is hang time multiplied by the gravitational force of the mouse [body mass (g) × 0.00980665 N/g × hang time (s)]^45^.

#### Accelerated rotarod test

Modified rotarod tests were conducted 2 weeks prior to sacrifice, as described previously^12^. Mice were conditioned on the rotarod (Ugo Basile, Varese, Italy) for 2 days; data were collected on the third day. For each trial, mice were placed on the rotarod for 1 min at 2 rpm; acceleration to 20 rpm took place over the next minute, and the latency to fall was analyzed.

#### Voluntary exercise

We used voluntary running wheel use to evaluate physical performance and endurance^46^. Mice were placed in cages with in-cage running wheels (10.16 cm diameter, Columbus Instruments, Columbus, OH, USA) with water and experimental diets for 24 h. Wheel rotations were monitored continuously in 1-min increments via magnetic switches interfaced to a computer. Wheel running tests were conducted every 4 weeks beginning at 24 weeks after diet initiation for young and middle-aged mice for a total of 7 tests. For aged males, wheel running tests were conducted every 4 weeks immediately after diet initiation. Distance travelled and speed were analyzed for each test. Mouse averages were calculated from each test period. All mice were returned to their home cages until the day of sacrifice.

### Cell culture

Mouse myoblast C2C12 cell line (ATCC CRL1772, Manassas, VA, USA) was cultured in Dulbecco’s Modified Eagle’s medium with 4.5 g/L glucose (DMEM, Lonza #12604Q, Walkersville, MD, USA) containing 10% heat-inactivated fetal bovine serum (FBS, Sigma #F4135), and 1% penicillin/streptomycin (Gibco Life Technologies #15070063) at 37 °C in a humidified atmosphere with 5% CO_2_. To differentiate C2C12 myoblasts from myotubes, 70% confluent myoblasts were switched to a differentiation medium consisting of DMEM containing 2% horse serum (HS, Sigma #12449C). Differentiation media was replaced every 1‒2 days until the presence of long multinucleated myotubes was observed, which occurred at 7‒10 days. Afterward, media were replaced with either CF or SAAR experimental media for 24 h. CF medium was a DMEM-based medium containing 100 mg/L cysteine, 31 mg/L cystine, and 15 mg/L methionine, supplemented with 2% HS. Eighty percent SAAR medium was created by diluting CF medium 5 times with custom DMEM that did not contain cysteine, cystine, or methionine (Gibco Life Technologies #21013024). The final concentrations of sulfur amino acids in SAAR medium were 20 mg/L cysteine, 6.2 mg/L cystine, and 3 mg/L methionine, as described previously^11^. After 24 h, cells were washed 3 times with ice-cold PBS followed by addition of Trizol or radioimmunoprecipitation assay (RIPA) buffer for RNA or protein analysis, respectively.

### Blood biochemical tests

On the day of sacrifice, blood was collected by retroorbital bleeding and glucose was measured using a handheld Abbott® Freestyle glucometer and test strips. Plasma was collected and ELISA kits were used to measure insulin (ALPCO Diagnostics, Salem, NH, USA), leptin, IGF-1, ADIPOQ (R&D Systems, Minneapolis, MN, USA), and FGF21 (Millipore Corp., Billerica, MA, USA).

### Gene expression analysis

Quadriceps femoris muscle (50 mg) was used for gene expression analysis by quantitative real-time PCR (qPCR) as previously described^7^. Briefly, 1 μg of RNA was reverse transcribed to cDNA using High-Capacity cDNA Reverse Transcription Kit (Life Technologies, Carlsbad, CA, USA), and qPCR was conducted in a StepOnePlus Real-Time PCR System using TaqMan primer–probe sets (Life Technologies; Supplementary Table S2). Gene expression was assessed by comparative CT (ΔΔCT) method using β-actin as reference.

### Mitochondrial DNA copy determination

Total DNA was isolated from ~ 25 mg of muscle tissues using a Qiagen DNeasy Blood and Tissue Kit (Qiagen, Germantown, MD, USA). The quantity and quality of DNA were determined by Nanodrop (Thermo Scientific, Waltham, MA, USA) and stored at 4 °C until used. Mitochondrial DNA was determined as described previously^47^. Briefly, 10 ng DNA was used to amplify *Ucp2* nuclear gene and 2 ng DNA for *CoxII* mitochondrial gene. Mouse primers used were forward primer Ucp2-F 5´-GCG TTC TGG GTA CCA TCC TAA C-3´; reverse primer Ucp2-R 5´- GCG ACC AGC CCA TTG TAG A—3´; forward primer CoxII-F 5´-TTT TCA GGC TTC ACC CTA GAT GA-3´; reverse primer CoxII-R 5´-GAA GAA TGT TAT GTT TAC TCC TAC GAA TAT G-3´; TaqMan probes for Ucp2—5´-CGC ACT GAG GGT CCA CGC AGC-3´and CoxII 5´-CAT GAG CAA AAG CCC ACT TCG CCA-3´ (BioSearch Technologies, Petaluma, CA,USA). Copy number was determined using 2 × 2^ΔCt^^48^. PCR reaction was carried out at 95 °C for 10 min followed by 40 cycles of 95 °C for 15 s and 60 °C for 1 min. The ratio of mtDNA to nuclear DNA reflects the concentration of mtDNA.

### SUnSET measurement of protein synthesis

The SUrface SEnsing of Translation (SUnSET) method was used to assess global protein synthesis^49^. For C2C12 myotubes, 10 μL of puromycin (50 μM; Research Products International, IL) was added to every cell culture well 5 min before collection. Upon collection, all wells were placed on ice and washed three times with ice-cold PBS. Cells were then scraped off the plate and collected in an Eppendorf tube, as described previously^50^.

### Citrate synthase and succinyl dehydrogenase activities

Citrate synthase and succinyl dehydrogenase activities were measured using commercially available kits (Sigma cat #MAK193 and #MAK197, respectively). Briefly, 10 mg muscle tissue were homogenized or a final volume 8 µl of cell lysates were processed using proprietary reagents. Samples were loaded onto a flat-bottomed 96-well plate, and absorbance was read at 412 nm using a SpectraMax plate reader (Molecular Devices, San Jose, CA, USA) which was maintained at 25 °C throughout the experiment. Absorbance was collected every 5 min for 20–40 min, and enzyme activity was calculated according to manufacturer’s protocol and reported as nmol/min/μl. Results were normalized to protein concentrations determined by micro bicinchoninic acid (BCA) assay, described in the following section (Western Blot Analysis).

### Western blot analysis

Muscle tissues (50 mg) were homogenized in ice-cold extraction buffer containing HEPES, Halt protease, and phosphatase inhibitors (Thermo Scientific) using Qiagen TissueLyser II. Protein content was determined by BCA protein assay kit (Thermo Scientific). Protein (15 μg) was electrophoresed in 4‒20% Mini-Protean TGX precast gels (Bio-Rad, Laboratories, Inc., Hercules, CA, USA), then transferred to polyvinylidene difluoride membranes (Bio-Rad). Membranes were blocked with 5% fat-free milk (or 5% bovine serum albumin) in TBS-0.1% Tween and then incubated with appropriate antibodies (Supplementary Table S3). To test multiple antibodies, the membrane was cut above and below the target molecular weight, and then incubated with the antibody overnight at 4 °C. Immunoblots were developed using a SuperSignal West Pico or Femto Chemiluminescence Kit (Thermo Scientific). Density of bands on membranes was analyzed using Image Lab software (Bio-Rad). Approximate size of specific bands was normalized to Ponceau S (Sigma) loaded in the same membrane.

### Statistical analyses

Statistics were completed using Prism 9 (GraphPad Software, La Jolla, CA, USA). Data are presented as means ± standard deviations (SD). For power analysis calculation to determine sample size, we follow published guidelines^51^. We have made assumptions on changes to achieve 80% power with α < 0.05. Fold change expression analyses were performed using two-tailed unpaired Student’s t-tests with *P* < 0.05 considered statistically significant. Comparisons within age and sex were analyzed using mixed-effect model 2-Way ANOVA with Sidak post-hoc comparisons with alpha threshold of 0.05 and 95% confidence level. Statistical significance is indicated as ^*^ for *P* < 0.05, ^**^ for *P* < 0.01, ^***^ for *P* < 0.001, and ^****^ for *P* < 0.0001.

### Supplementary Information

Supplementary Information.

## Data Availability

The data that support the findings of this study are available on request from the corresponding author.
