# Supplementary material for: Physical activity of mice on dietary sulfur amino acid restriction is influenced by age of diet initiation and biological sex
Source: Sci Rep. 2023 Nov 23;13:20609. doi: 10.1038/s41598-023-47676-7 (PMC10667228; doi:10.1038/s41598-023-47676-7)
Supplement: Supplementary file 1 — Supplementary Information. [file 41598_2023_47676_MOESM1_ESM.pptx]

## Slide 1
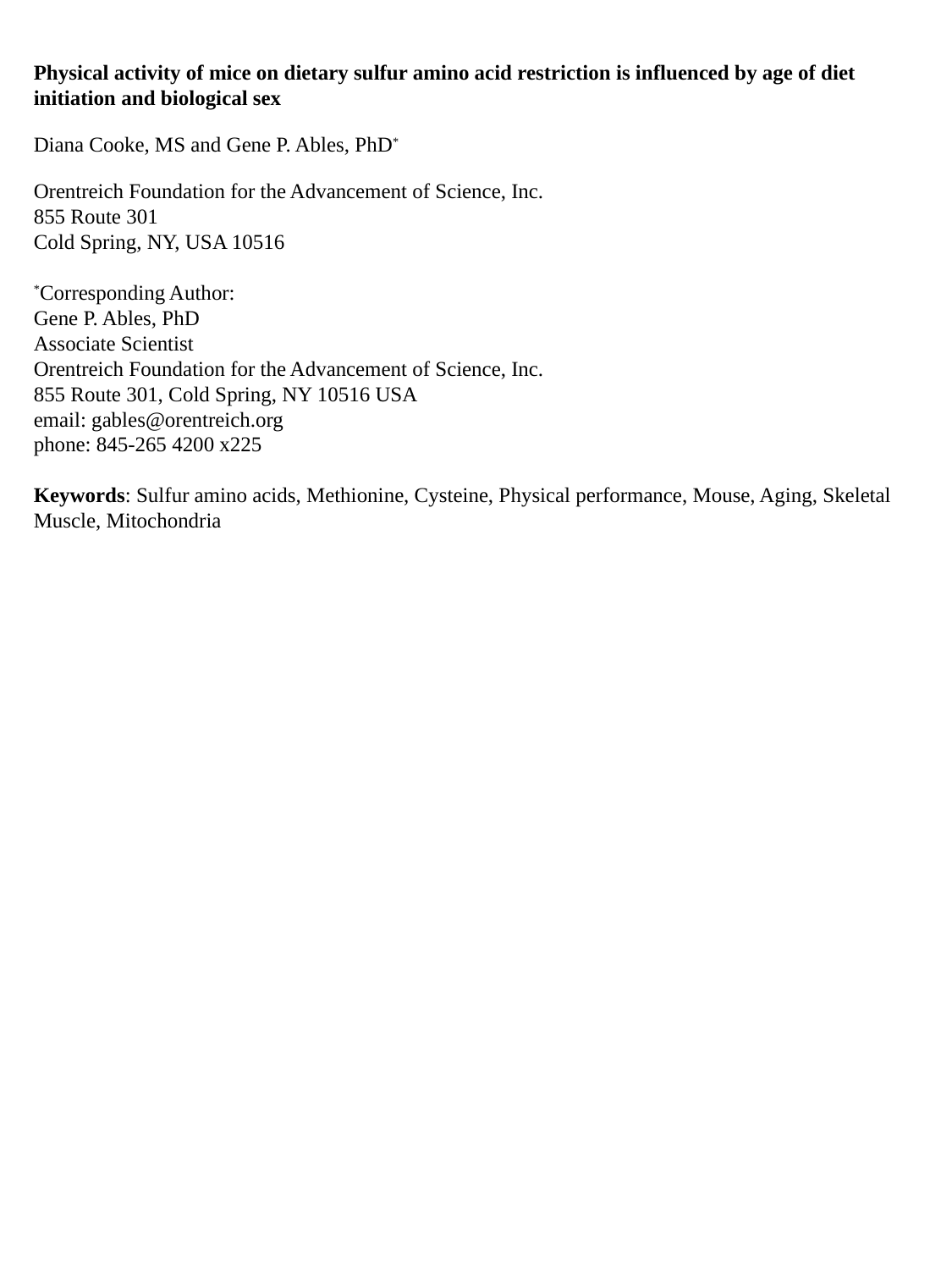

Physical activity of mice on dietary sulfur amino acid restriction is influenced by age of diet initiation and biological sex
Diana Cooke, MS and Gene P. Ables, PhD*
Orentreich Foundation for the Advancement of Science, Inc.
855 Route 301
Cold Spring, NY, USA 10516
*Corresponding Author:
Gene P. Ables, PhD
Associate Scientist
Orentreich Foundation for the Advancement of Science, Inc.
855 Route 301, Cold Spring, NY 10516 USA
email: gables@orentreich.org
phone: 845-265 4200 x225
Keywords: Sulfur amino acids, Methionine, Cysteine, Physical performance, Mouse, Aging, Skeletal Muscle, Mitochondria

## Slide 2
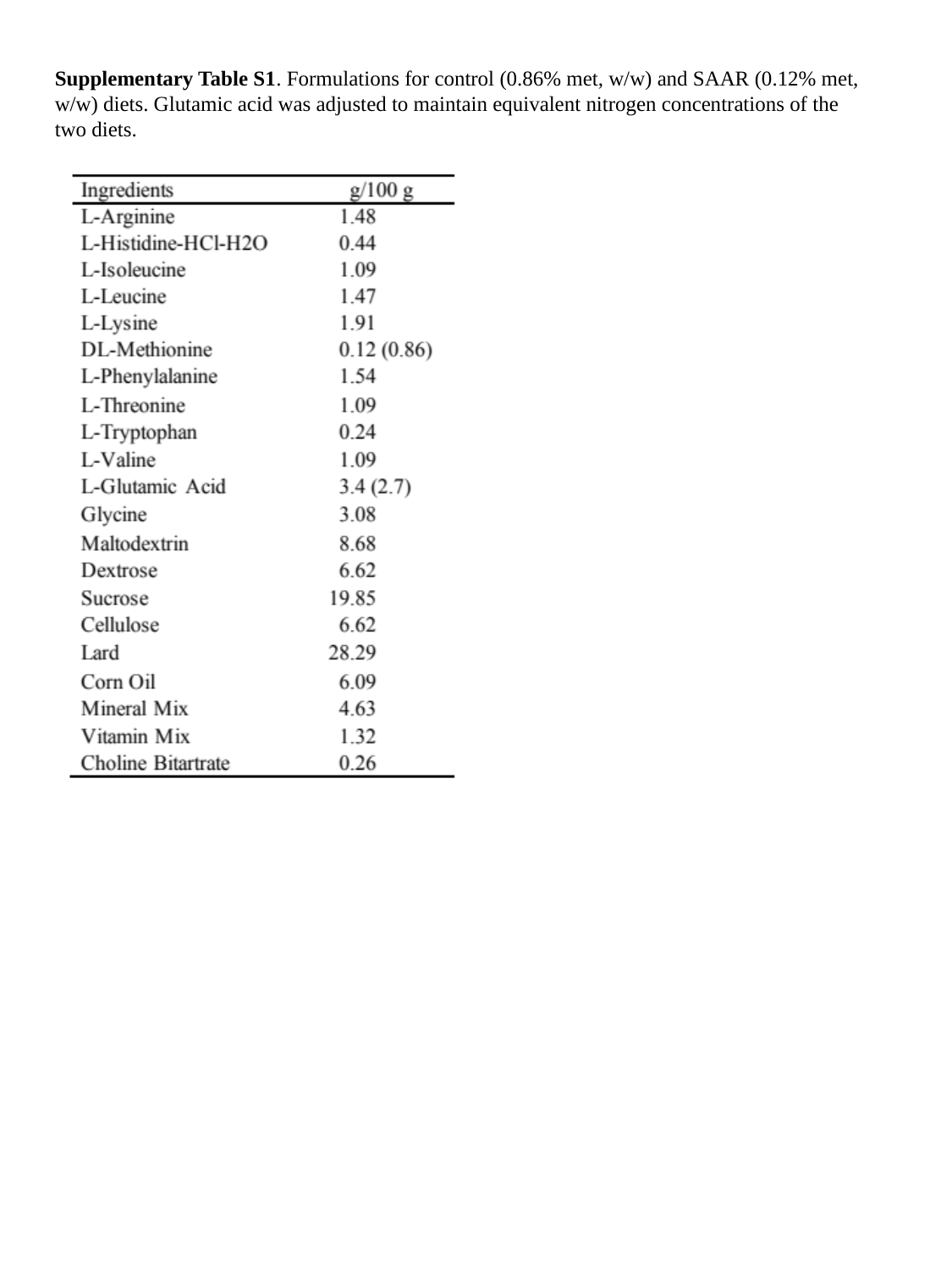

Supplementary Table S1. Formulations for control (0.86% met, w/w) and SAAR (0.12% met, w/w) diets. Glutamic acid was adjusted to maintain equivalent nitrogen concentrations of the two diets.

## Slide 3
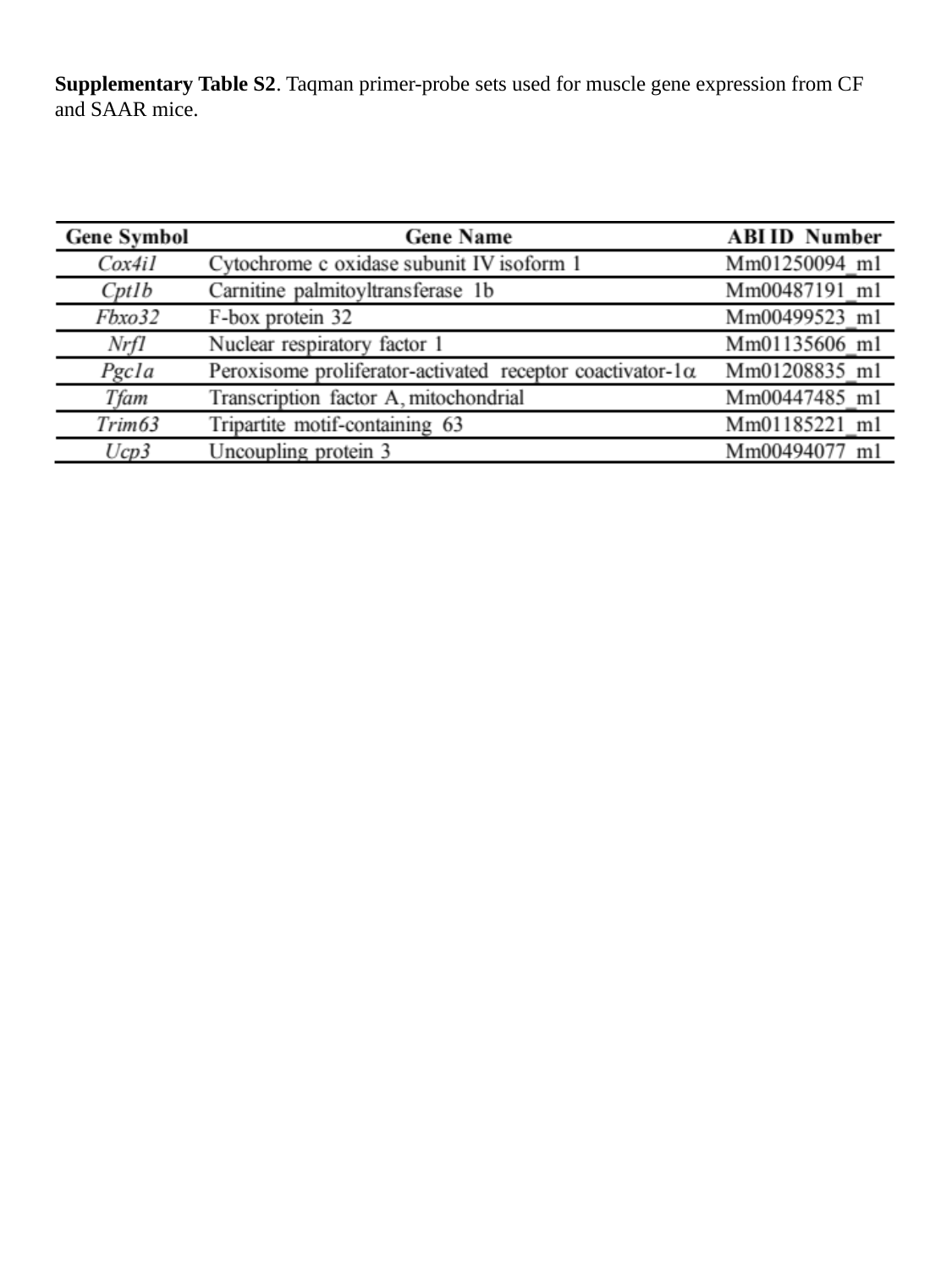

Supplementary Table S2. Taqman primer-probe sets used for muscle gene expression from CF and SAAR mice.

## Slide 4
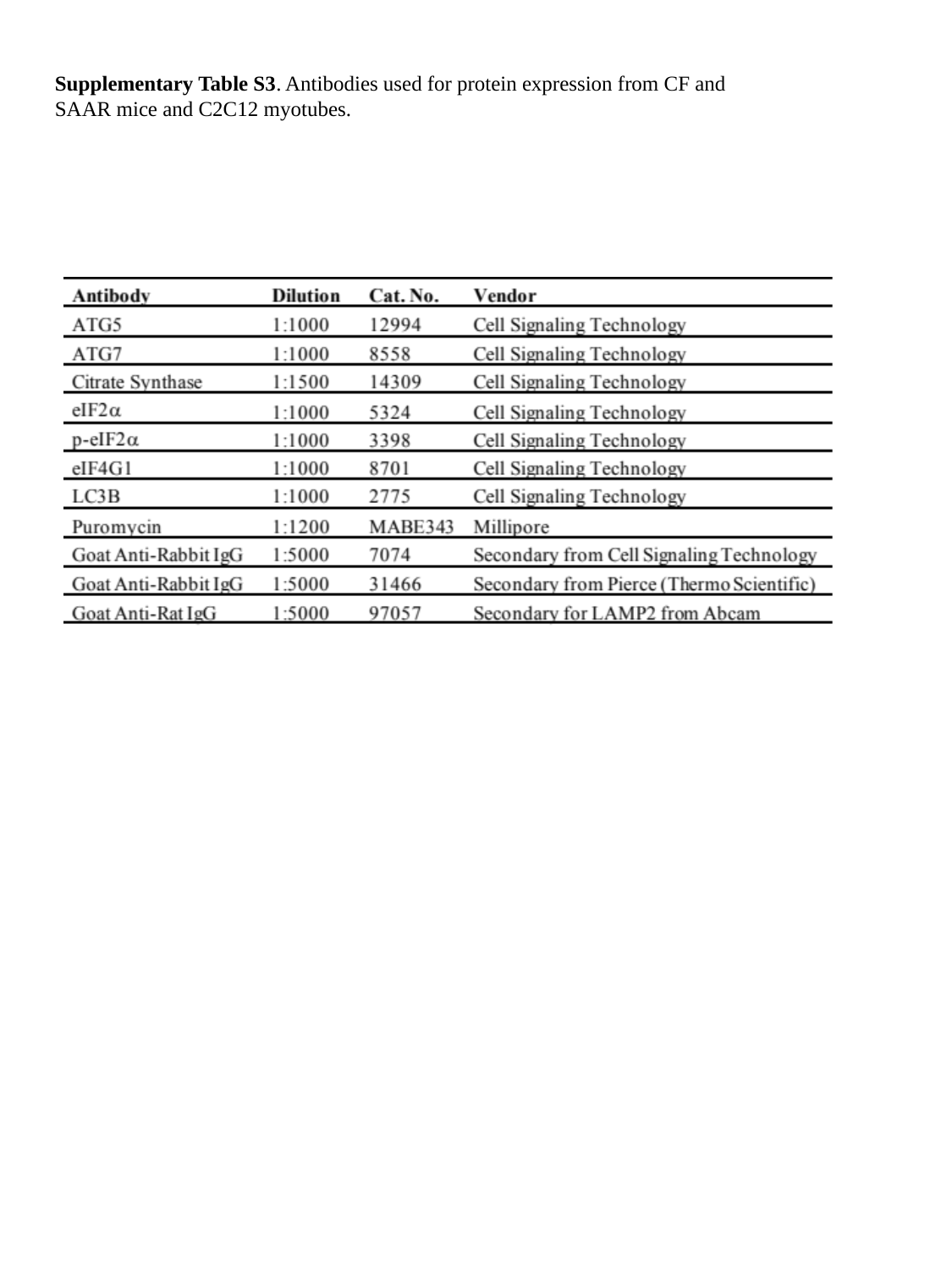

Supplementary Table S3. Antibodies used for protein expression from CF and SAAR mice and C2C12 myotubes.

## Slide 5
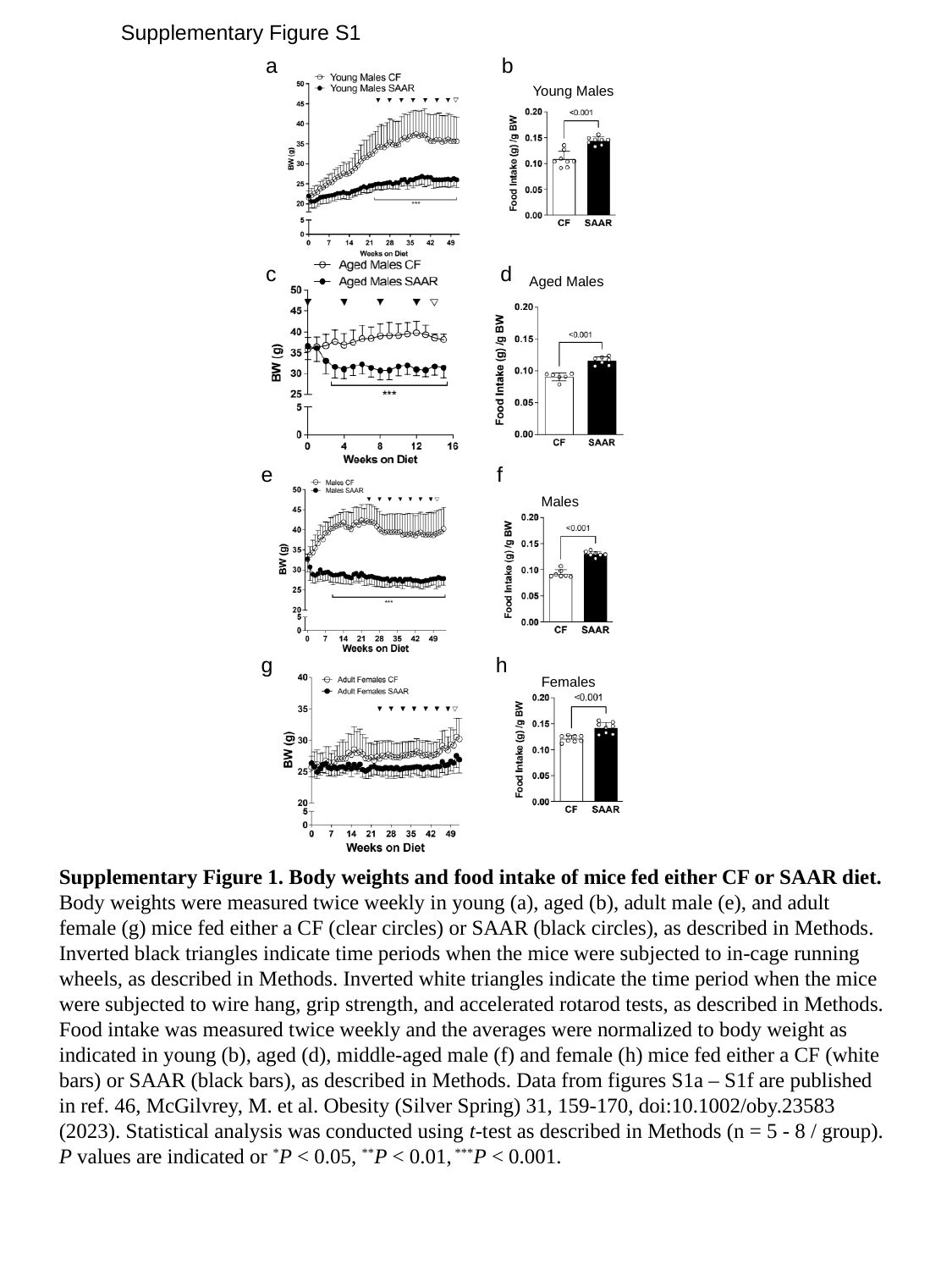

Supplementary Figure S1
a
b
Young Males
c
d
Aged Males
e
f
Males
g
h
Females
Supplementary Figure 1. Body weights and food intake of mice fed either CF or SAAR diet. Body weights were measured twice weekly in young (a), aged (b), adult male (e), and adult female (g) mice fed either a CF (clear circles) or SAAR (black circles), as described in Methods. Inverted black triangles indicate time periods when the mice were subjected to in-cage running wheels, as described in Methods. Inverted white triangles indicate the time period when the mice were subjected to wire hang, grip strength, and accelerated rotarod tests, as described in Methods. Food intake was measured twice weekly and the averages were normalized to body weight as indicated in young (b), aged (d), middle-aged male (f) and female (h) mice fed either a CF (white bars) or SAAR (black bars), as described in Methods. Data from figures S1a – S1f are published in ref. 46, McGilvrey, M. et al. Obesity (Silver Spring) 31, 159-170, doi:10.1002/oby.23583 (2023). Statistical analysis was conducted using t-test as described in Methods (n = 5 - 8 / group). P values are indicated or *P < 0.05, **P < 0.01, ***P < 0.001.

## Slide 6
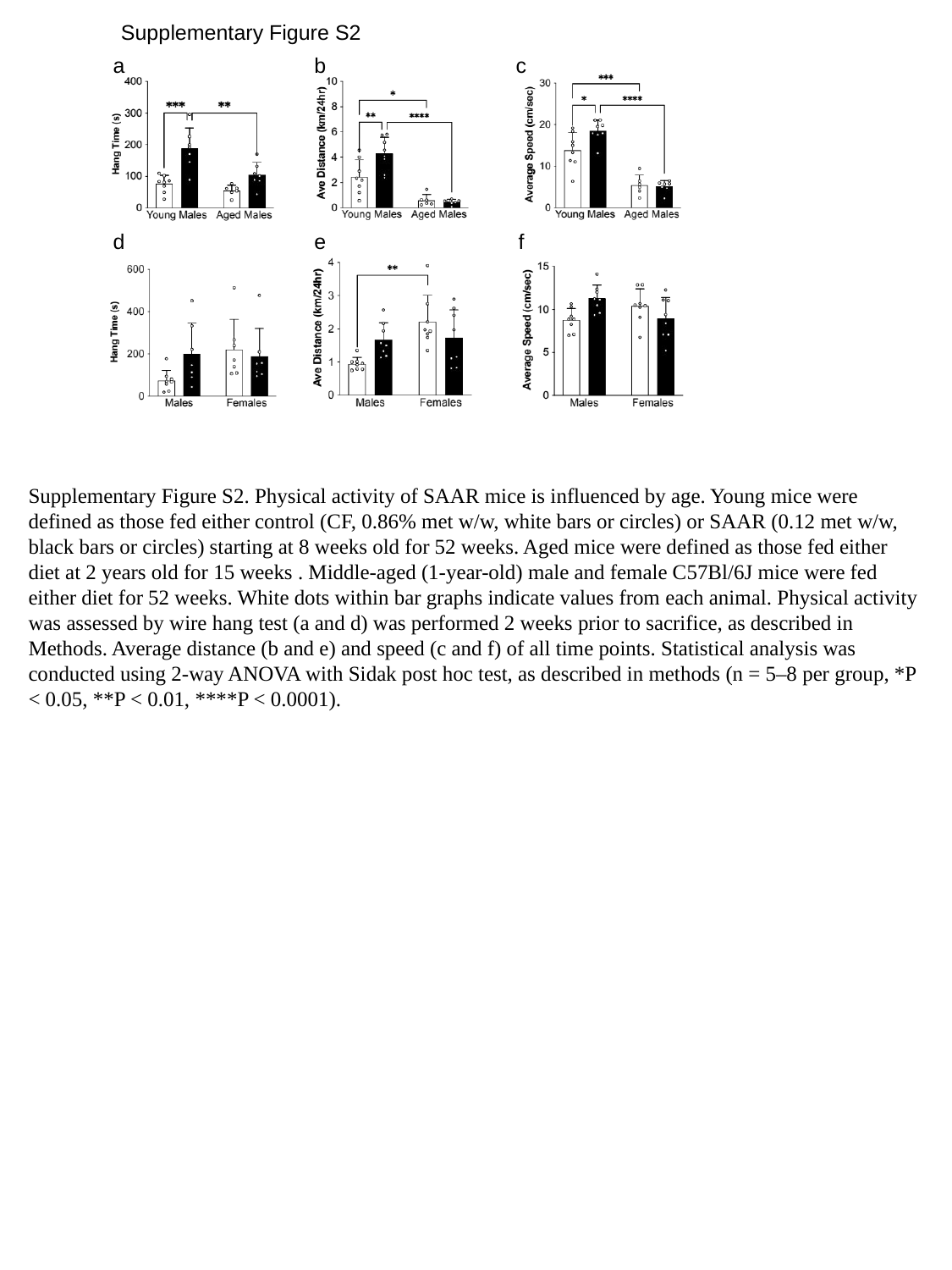

Supplementary Figure S2
a
b
c
d
e
f
Supplementary Figure S2. Physical activity of SAAR mice is influenced by age. Young mice were defined as those fed either control (CF, 0.86% met w/w, white bars or circles) or SAAR (0.12 met w/w, black bars or circles) starting at 8 weeks old for 52 weeks. Aged mice were defined as those fed either diet at 2 years old for 15 weeks . Middle-aged (1-year-old) male and female C57Bl/6J mice were fed either diet for 52 weeks. White dots within bar graphs indicate values from each animal. Physical activity was assessed by wire hang test (a and d) was performed 2 weeks prior to sacrifice, as described in Methods. Average distance (b and e) and speed (c and f) of all time points. Statistical analysis was conducted using 2-way ANOVA with Sidak post hoc test, as described in methods (n = 5–8 per group, *P < 0.05, **P < 0.01, ****P < 0.0001).

## Slide 7
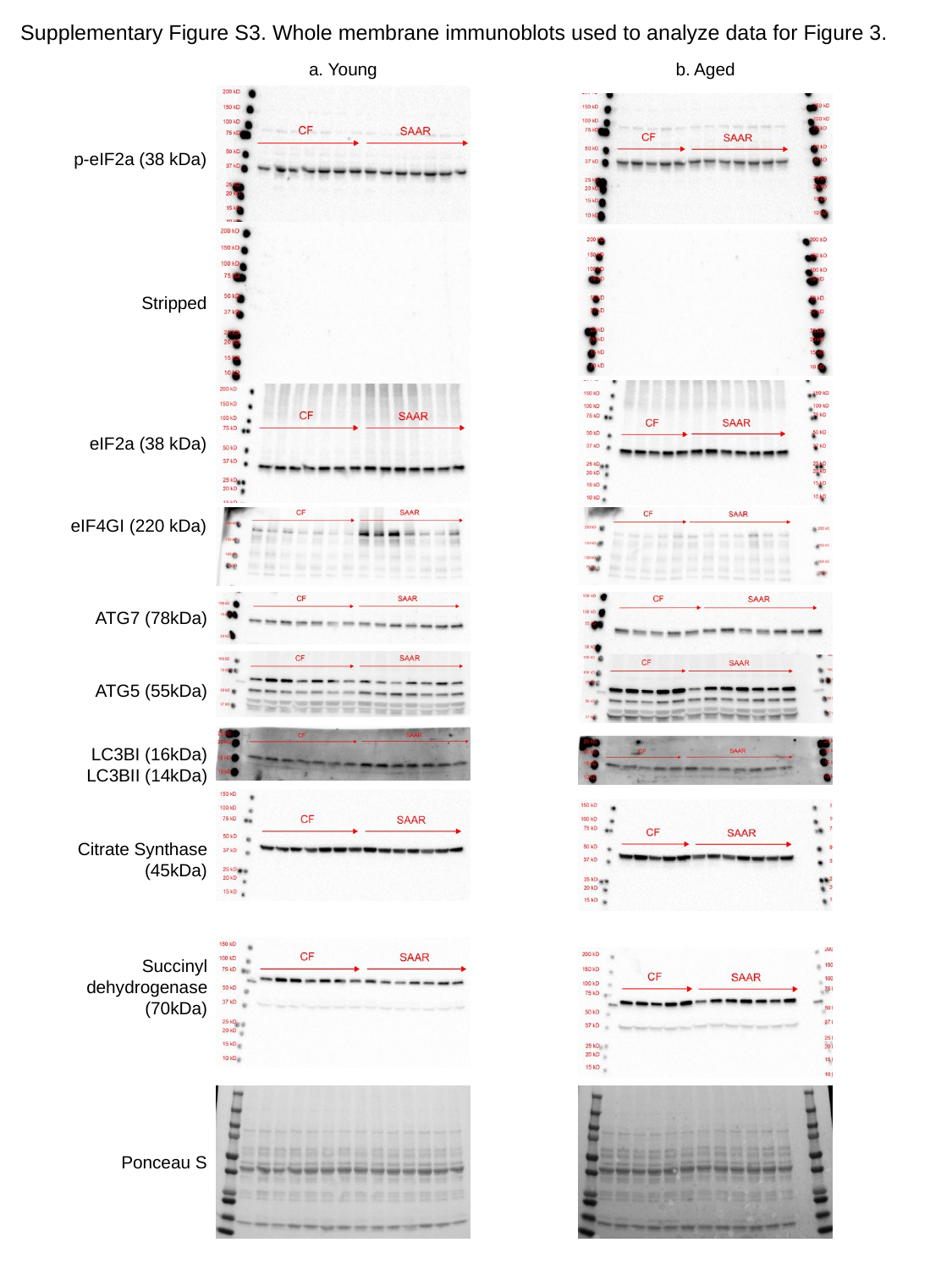

Supplementary Figure S3. Whole membrane immunoblots used to analyze data for Figure 3.
a. Young
b. Aged
p-eIF2a (38 kDa)
Stripped
eIF2a (38 kDa)
eIF4GI (220 kDa)
ATG7 (78kDa)
ATG5 (55kDa)
LC3BI (16kDa)
LC3BII (14kDa)
Citrate Synthase (45kDa)
Succinyl dehydrogenase (70kDa)
Ponceau S

## Slide 8
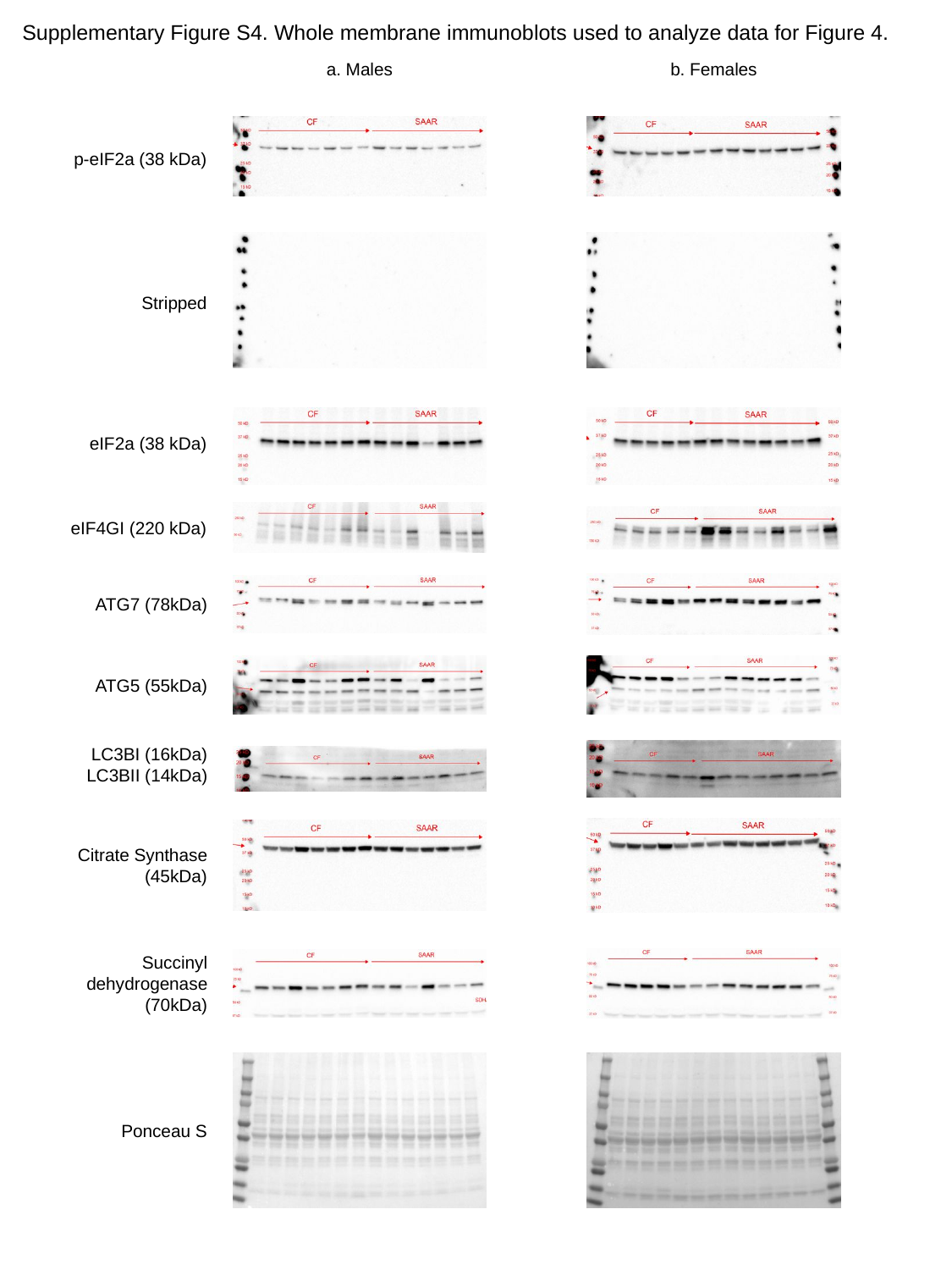

Supplementary Figure S4. Whole membrane immunoblots used to analyze data for Figure 4.
a. Males
b. Females
p-eIF2a (38 kDa)
Stripped
eIF2a (38 kDa)
eIF4GI (220 kDa)
ATG7 (78kDa)
ATG5 (55kDa)
LC3BI (16kDa)
LC3BII (14kDa)
Citrate Synthase (45kDa)
Succinyl dehydrogenase (70kDa)
Ponceau S

## Slide 9
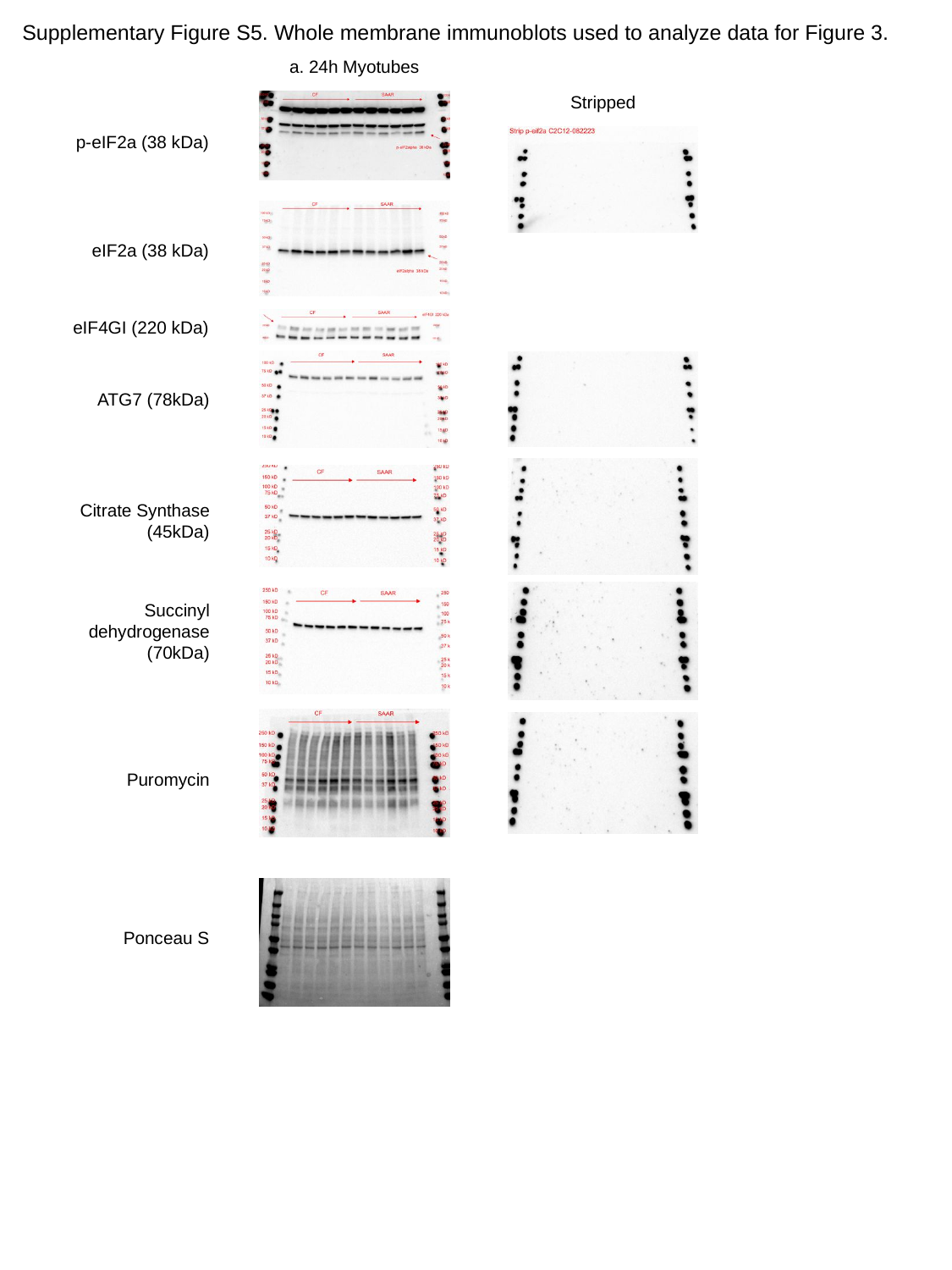

Supplementary Figure S5. Whole membrane immunoblots used to analyze data for Figure 3.
a. 24h Myotubes
Stripped
p-eIF2a (38 kDa)
eIF2a (38 kDa)
eIF4GI (220 kDa)
ATG7 (78kDa)
Citrate Synthase (45kDa)
Succinyl dehydrogenase (70kDa)
Puromycin
Ponceau S
